# A rare presentation of a common carotid artery occlusion

**DOI:** 10.3205/oc000228

**Published:** 2023-11-07

**Authors:** Amber Demeuleneere, Julie Lambert, Jelle Demeestere, Robin Lemmens, Inge Fourneau, Sabrina Houthoofd, Pieter-Paul Schauwvlieghe, Julie Jacob, Catherine Cassiman

**Affiliations:** 1 Ophthalmology Department, University Hospitals Leuven, Belgium; 2 Radiology Department, University Hospitals Leuven, Belgium; 3 Neurology Department, University Hospitals Leuven, Belgium; 4 Vascular Surgery Department, University Hospitals Leuven, Belgium

**Keywords:** common carotid artery occlusion, visual loss, elevation paresis, internal carotid artery

## Abstract

**Background:**

A common carotid artery occlusion (CCAO) is very rare and the clinical features of CCAO have rarely been described. Since the blood supply of the eye and orbit is derived from the internal carotid artery, a CCAO may present with various ophthalmological symptoms, ranging from incidental findings to complete visual loss but also other neuro-ophthalmological abnormalities.

**Case report:**

A 61-year-old woman presented with acute monocular vision loss and an elevation deficit of the right eye. Fluorescein angiography showed delayed filling of both the retinal and choroidal vasculature, without occlusion/embolisms of the retinal arteries. Vascular imaging showed a right CCAO.

**Conclusion:**

CCAO has a variable presentation. In patients with acute unilateral visual loss a CCAO should be considered, especially when ocular motility deficits are present. Fluorescein angiography examination can aid in the localization and diagnosis of the vascular insult. Urgent referral for a systemic work-up is essential.

## Introduction

Acute common carotid artery occlusion (CCAO) may remain asymptomatic or present with a variety of symptoms. Since the blood supply of the eye and orbit is derived from the internal carotid artery, a CCAO can present with ischemic ocular symptoms, as shown in this case report. 

## Case description

A 61-year-old Caucasian woman presented herself at the ophthalmology department of the University Hospitals Leuven, Belgium. She had woken up that morning with a frontal, right-sided headache, and a complete loss of vision of the right eye. She also mentioned that she could not feel whether the right eye was open or not. She had a past medical history of diffuse large B cell lymphoma treated with chemotherapy (R-CHOP) one year ago. She had no known history of cardiovascular events, a positive history for smoking, with a present use of 5 cigarettes a day. There was no use of medication, except for occasional use of Zolpidem. 

**Figure 1:**
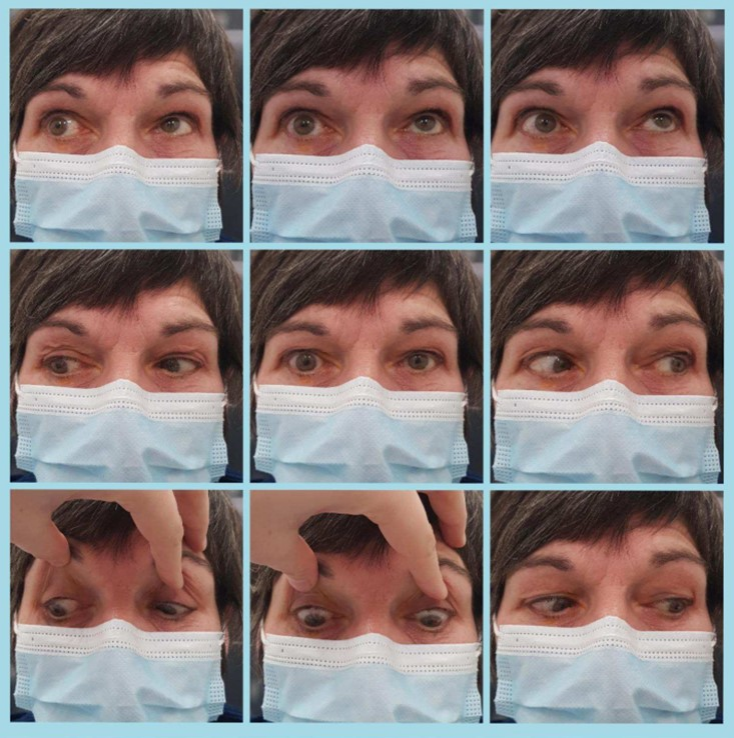
Nine-gaze-picture at presentation. As seen in the three top pictures there is an elevation deficit of the right eye.

Ophthalmological examination showed the best corrected visual acuity was light perception without localization for the right eye (OD) and 1.0 for the left eye (OS). Examination of the eye movements showed a unilateral elevation deficit of the right eye (Figure 1 (Fig. 1)). The intra-ocular pressure measured with Goldmann applanation tonometry was 9.0 (OD) and 12.0 (OS). Slit lamp microscope examination of both anterior segments showed no abnormalities, as well as dilated fundoscopy. There was no sign of anisocoria, there was however a relative afferent pupillary defect (RAPD) of the right eye. There was no dilatation lag. There was no ptosis and normal eyelid opening. Sensory supply of the V1-V3 area was intact, as was the corneal reflex. There was no temporal artery tenderness to palpation. There was no proptosis; Hertel exophthalmometry showed a symmetrical finding of 14 mm for each eye. 

Laboratory findings including complete blood count, C-reactive protein, sedimentation rate, comprehensive metabolic panel, immunology auto-antibodies (antinuclear antibodies and antineutrophil cytoplasmic antibodies), bacterial serology (borrelia, toxoplasma, *Treponema pallidum*) and viral serology (cytomegalovirus, Epstein-Barr virus, HIV, varicella-zoster virus), were all negative and/or unremarkable, with exception of an elevated angiotensin-converting enzyme count of 89 U/l (8-52 U/l). 

**Figure 2:**
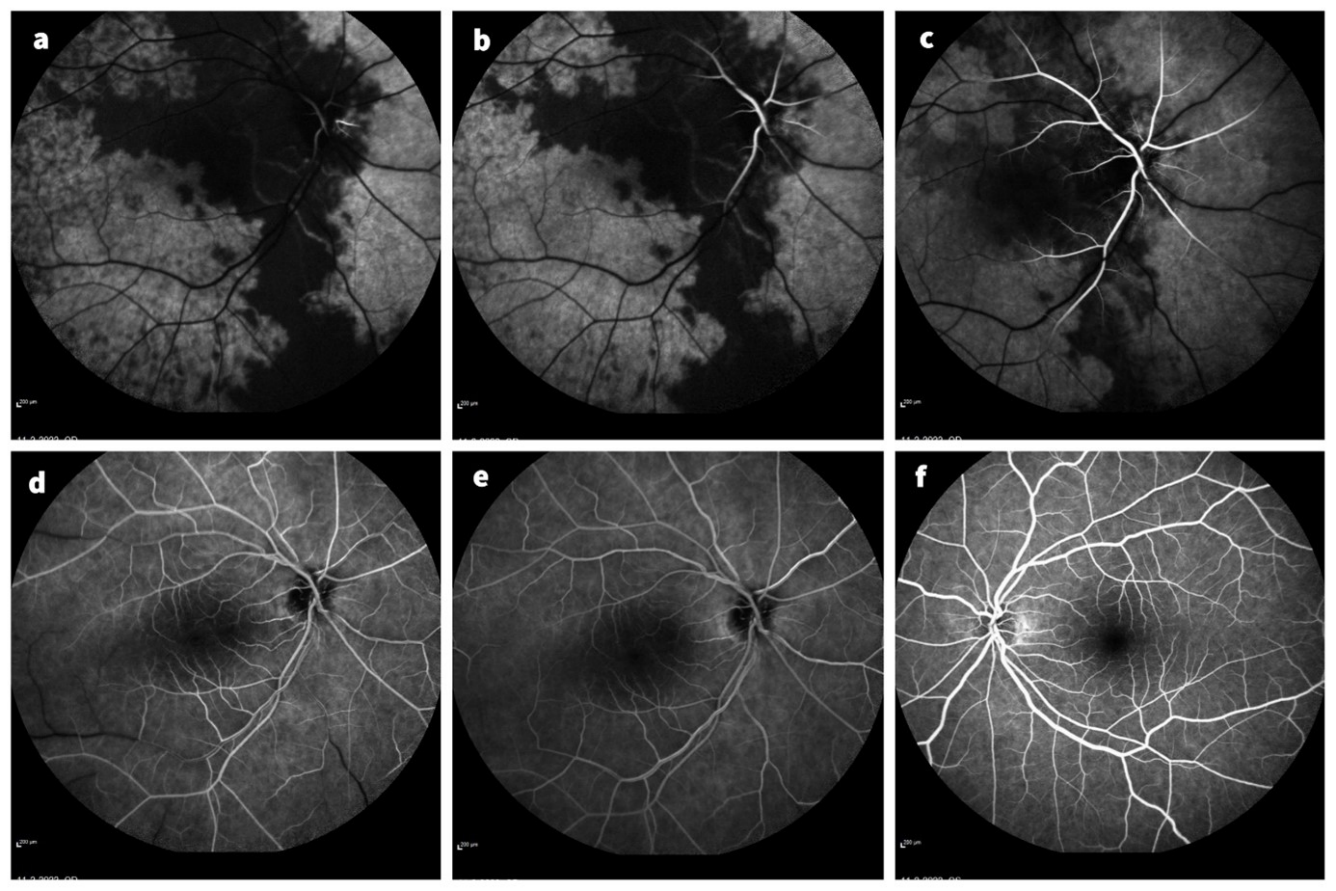
Fluoresceine angiography at presentation, a to e show the images of the right eye, f shows the image of the left eye. a: 44 seconds after fluorescein injection the fluorescein becomes visible in the retinal vessels, the choroidal filling is still incomplete. b: 52 seconds after injection. c: 1 minute 21 seconds after injection. d: 3 minutes 37 seconds after injection, there is still no filling of the midperipheral retinal vessels as seen in the inferior and temporal vessels. e: 5 minutes 37 seconds after injection, still an incomplete peripheral filling (not seen in the picture). f: 1 minute 9 seconds after injection, the left eye showed a normal filling of the retinal and choroidal vessels.

Fluoresceine angiography was performed at presentation. It showed a remarkably slow and incomplete filling of both the choroidal and retinal vasculature of the right eye. Retinal filling only began one minute after intravenous injection of the fluorescein dye. Eight minutes after injection there was still an incomplete filling of the peripheral retinal vessels (Figure 2 (Fig. 2)).

**Figure 3:**
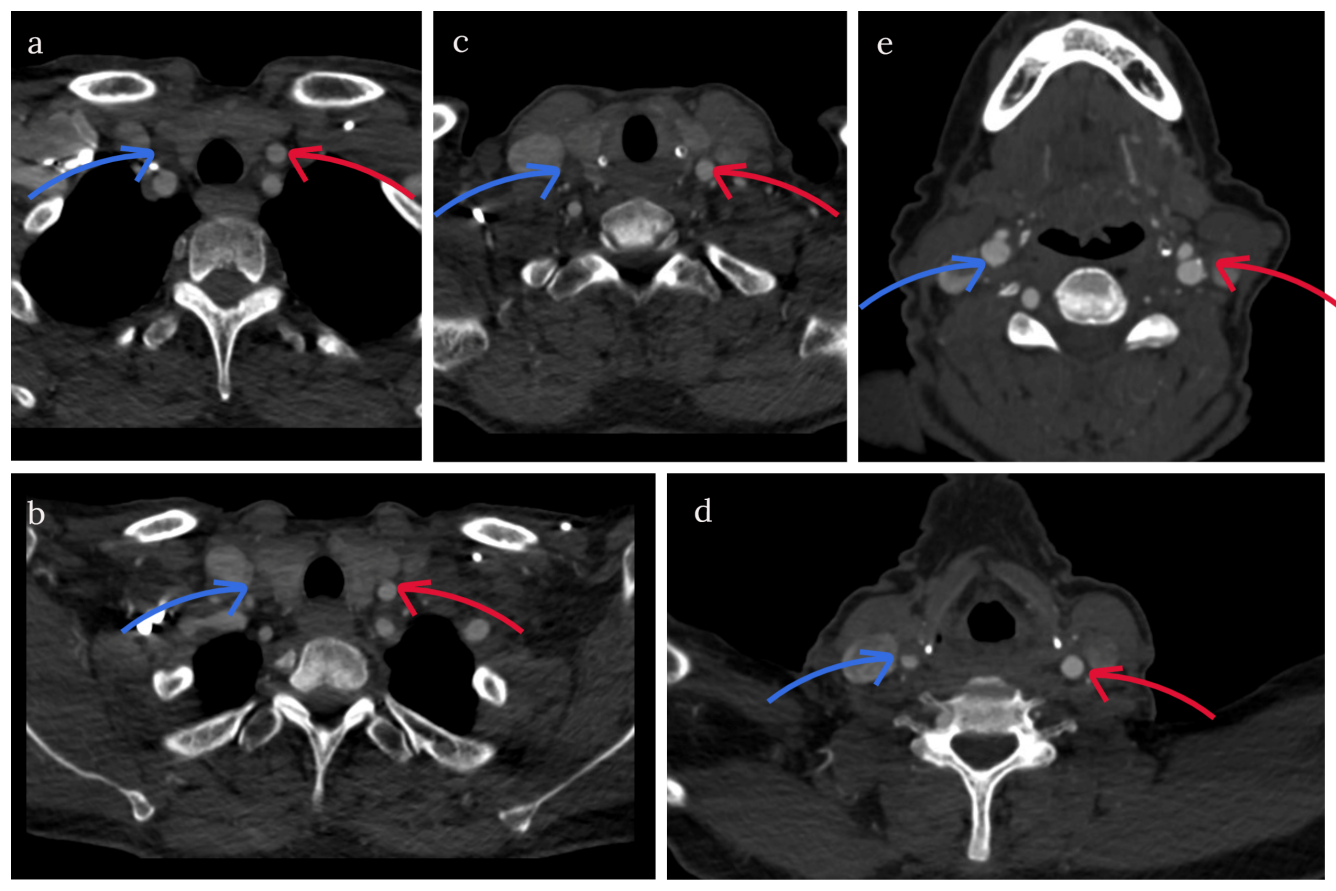
CT angiography images at presentation: a long segmentary occlusion of the right common carotid artery. The blue arrow shows the right CCA, the red arrow shows the left CCA. In picture a, b and c there is a complete occlusion of the right CCA. Picture d shows a partial filling of the right CCA, just before the bifurcation. Picture e shows a complete filling of the right CCA/ICA at the level of the bifurcation.

The patient was urgently referred to the colleagues at the neurology department. Brain imaging showed no abnormalities. Computed tomography (CT) angiography of head and neck vessels revealed a proximal occlusion of the right common carotid artery (ACC), with distal filling through collaterals and a narrow internal carotid artery (ACI) (Figure 3 (Fig. 3)), most likely because of low flow. Magnetic resonance imaging (MRI) of the brain showed no sign of embolism or recent ischemia. The patient was admitted to the stroke unit a dual and antiplatelets therapy was started.

One week and 4 months after the initial event, a slight gradual improvement in visual acuity of the right eye to counting fingers and a partial recuperation of elevation of the right eye was found. Optic coherence tomography showed atrophy of the retinal layers four months after presentation.

## Discussion

**Figure 4:**
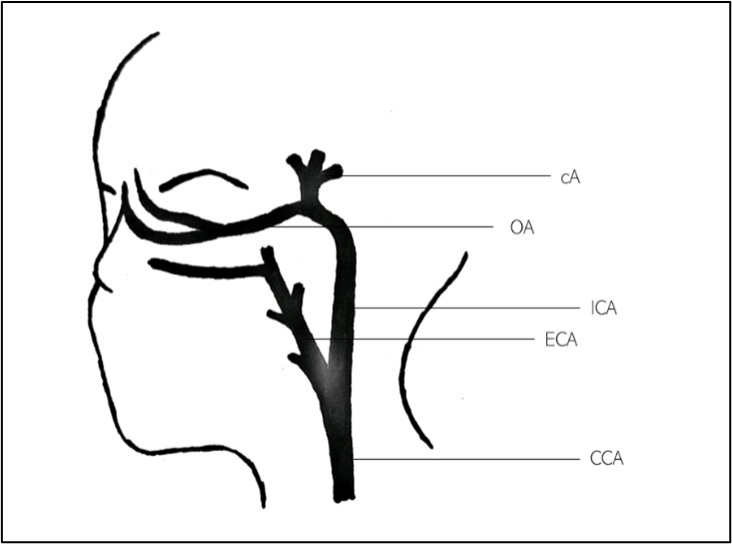
Simplified view of the carotid arteries. CCA: common carotid artery, ECA: external carotid artery, ICA: internal carotid artery, OA: ophthalmic artery, cA: cerebral arteries

We present a case of acute unilateral visual loss and eye movement abnormalities after CCAO. The blood supply to the eye derives from the ICA, as seen in Figure 4 (Fig. 4), and hence, a CCAO can present with ischemic ocular symptoms (e.g. amaurosis fugax, branch or central retinal artery occlusion, ophthalmic artery occlusion, or ocular ischemic syndrome) [1].

A stenosis of the carotid artery due to atherosclerotic disease usually arises from the internal carotid artery (ICA). The incidence of a steno-occlusive disease of the common carotid artery (CCA) is very low; the incidence in stroke cases has been reported as 1–5% in literature [2]. In a large case series, where 6,415 patients underwent color duplex imaging for suspected carotid artery disease (symptomatic carotid disease, symptom-free carotid bruit, precardiac and preaortic surgical evaluation and postcarotid endarterectomy), Parthenis et al. reported an incidence of CCAO of 0.54% [3]. A similar case series by Chang et al. [2] reported an incidence of CCAO of 0.24%, among the 5,400 duplex ultrasonograms studied for detecting suspected carotid artery disease. Since not all patients with CCAO are symptomatic, there is probably an underestimation of the incidence of CCAO [4].

Two types of CCAO are described. Type I: Both CCA and ICA are occluded and the ischemic events are due to hemodynamical background. Type II: CCA is occluded but ICA remains patent because of the supply of extracranial collateral flow [4], as was the case in our patient.

CCAO has a variable presentation. A study by Hoya et al. [5] investigated 12 patients with a CCA stenosis of whom 8 were symptomatic. Of the symptomatic patients, 3 presented with amaurosis fugax (AF) as sole complaint, 1 patient presented with a combination of AF and hemiparesis, and 4 other patients had a hemiparesis. The amount of CCA stenosis in the patients with AF was more than 75% in this series. In comparison with the reported frequency of AF in patients with ICA stenosis, the current study found a very high (50%) rate of AF in symptomatic CCA stenosis, suggesting that patients with CCA steno-occlusive lesions may be more susceptible to AF than those with ICA lesions [5]. They suggest that CCA stenosis can cause amaurosis fugax via two mechanisms. The first mechanism, the most common one, is thrombo-embolic. Emboli originating from a carotid atherosclerotic lesion enter into the ophthalmic circulation, causing sudden partial or complete monocular visual loss lasting seconds to minutes [5]. The second mechanism is the hemodynamic mechanism. This mechanism suggests that a change in posture, exercise, or exposure to bright light can induce retinal vascular insufficiency in patients with carotid steno-occlusive disease [5], [6].

When a CCAO affects the eye, it most often causes visual loss. Depending on whether there is a chronic or acute ischemia, the visual loss can be gradual or very sudden [7]. The degree of visual loss, or the amount of remaining visual acuity can range from light perception to 1.0. Ischemia of the intraorbital structures may rarely be observed [8] because of a number of potential collateral channels between the external and internal carotid system. One or more of these collaterals might be sufficiently large to take over the blood supply to the orbital structures at time of ICA occlusion in the majority of individuals [9]. Man et al. reported a case with an isolated pupil-involving third-nerve palsy due to CCAO [10]. Bogousslavsky et al. [8] described a patient with an isolated orbital infarction due to a common carotid artery occlusion, including visual loss, ophthalmoplegia, a non-reactive dilated pupil, corneal anesthesia and orbital pain. The pattern of ophthalmoplegia, with relative sparing of adduction, was more compatible with a muscle than a nerve dysfunction [8]. The non-reactive dilated pupil, corneal anesthesia and orbital pain, however, suggested nerve dysfunction of the ocular motor nerves and ophthalmic division of the trigeminal nerve [8].

In most cases atherosclerosis is the underlying cause of the occlusion. It occurs more often in the elderly and men are affected more often than women, which is related to the higher incidence of cardiovascular disease, the underlying morbidity, in males [7]. In patients with a poor collateral circulation between the carotid arteries, the risk of symptoms/damage is higher. Since it is associated with atherosclerosis, patients usually have other related co-morbidities (cardiovascular disease, hypertension, diabetes…) [7].

In our patient it remains unclear whether the underlying mechanism was hypoperfusion or embolization. After multidisciplinary deliberation it was suggested that an embolism had occurred at the time of CCAO. There was, however, no sign of embolization on the MRI to confirm this suggestion. Remarkable in this case is the combination of visual loss and elevation deficit. Presumably the optic nerve and superior rectus muscle were damaged in our patient because the collaterals who were meant to supply these structures were insufficient at the time of occlusion, causing the sudden vision loss and the elevation deficit of the right eye.

The diagnosis of ocular ischemia as the underlying cause of the visual loss can be confirmed using fluorescein angiography. The prolonged arm-to-choroid and arm-to-retina circulation time is a frequent sign. The normal arteriovenous retinal filling time is approximately 5 seconds, but in the affected eyes it may be 1 minute or longer, as seen in our patient [7], [11], [12]. Absence of retinal arterial stasis and choroidal filling defects detected by fluorescein angiography in diabetic retinopathy and CRVO is an important feature distinguishing these two conditions from ocular ischemia secondary to CCAO [13]. Depending on the clinical presentation, the differential diagnosis could include other vascular pathologies like central retinal vein occlusion, hyperviscosity syndromes and auto-immune vasculopathies [13].

The treatment of a patient with CCAO should be multidisciplinary, including cardiologist, neurologist, primary care physician, ophthalmologist and vascular surgeon. The treatment should be causal, with equal attention to secondary prevention of new events (i.e., restoration of the carotid artery patency and prevention of its stenosis) [7]. Carotid artery endarterectomy (CEA) is a surgical method used in the treatment of carotid artery stenosis [14]. It is effective in symptomatic carotid artery stenosis of 70–90% and in asymptomatic stenosis of at least 60%. Carotid artery stenting is an alternative treatment in case of stenosis. However, in cases of complete occlusion, an intervention to re-open the carotid artery might cause the thrombus to travel distally to other large arteries [7]. In our case arterial by-pass surgery could have been performed, but due to the presence of an adequate collateral circulation a conservative approach with systemic antiplatelet treatment was started. Systemic treatment includes antiplatelet agents, treatment of hypertension, atherosclerosis, diabetes mellitus and coronary heart disease. Appropriate diet, cessation of smoking, and physical activity play a very important role in secondary prevention [7].

## Conclusion

A patient with a CCAO can present at first to the ophthalmologist. The incidence of CCAO is not well-documented because of variable presentations. There is no complete record of the affected patients with this entity because of a significant part being asymptomatic in nature [4]. Our patient presented with a combination of acute monocular vision loss and an acute elevation paresis of the right eye. Performance of a fluoresceine examination showed a delayed filling of both the retinal and choroidal vasculature, suggesting an underlying vascular cause.

In this case an urgent referral to the department of neurology is essential. Imaging of the brain (CT angiography and MRI) were performed on the same day, showing a complete occlusion of the common carotid artery. Because of the presence of an adequate collateral circulation, a conservative approach with systemic treatment including antiplatelet agents was preferred.

To our knowledge this is the first report of a patient with a CCAO who presented with both profound visual loss and an elevation paresis. Since patients with CCAO are also at higher risk of developing other cardiovascular diseases [4], establishing the diagnosis is essential with respect to visual prognosis, but also with regard to the patients’ general health and overall survival [12]. Ocular signs may be the first manifestation of carotid artery disease [15], therefore, ophthalmologists have an important role in early diagnosis and referral for systemic work-up of patients.

## Notes

### Consent for publication

Hereby we confirm that the patient in this case report has given consent to participate as well as consent to publish all the data and images used in this case report. 

### Competing interests

The authors declare that they have no competing interests.
